# CRISPR-on-Chip for Point-of-Care Diagnostics

**DOI:** 10.1021/acsnano.5c19771

**Published:** 2026-01-13

**Authors:** Nazente Atceken, Alptekin Kahya, Defne Yigci, Savas Tasoglu

**Affiliations:** 1 School of Biomedical Sciences and Engineering, 52979 Koç University, Istanbul 34450, Turkey; 2 Koç University Translational Medicine Research Center (KUTTAM), 52979 Koç University, Istanbul 34450, Turkey; 3 School of Medicine, Koç University, Istanbul 34450, Turkey; 4 Department of Medicine, Solna (MedS), Division of Dermatology and Venereology, Karolinska Institutet, Stockholm 17177, Sweden; 5 Centre for Molecular Medicine, Karolinska University Hospital, Stockholm 17177, Sweden; 6 Department of Mechanical Engineering, 52979 Koç University, Istanbul 34450, Turkey; 7 Koç University Is Bank Artificial Intelligence Lab (KUIS AI Lab), 52979 Koç University, Istanbul 34450, Turkey; 8 Koç University Arçelik Research Center for Creative Industries (KUAR), 52979 Koç University, Istanbul 34450, Turkey; 9 Boğaziçi Institute of Biomedical Engineering, Boğaziçi University, Istanbul 34684, Turkey

**Keywords:** CRISPR-based diagnostic, microfluidics, point-of-care (PoC), CRISPR-on-chip technology

## Abstract

CRISPR-based diagnostic platforms have gained significant momentum in recent years, enabling highly sensitive and specific detection of pathogens and diseases. Due to their practical benefits, these platforms have become widely adopted in point-of-care (PoC) applications. CRISPR-on-chip technology integrates CRISPR-Cas platforms with diverse microfluidic systems, allowing scalability and portable, real-time, and precise biomolecule detection. This approach enhances diagnostic accuracy, reduces processing times, and minimizes the need for complex laboratory infrastructures, unlike in conventional diagnostics. Using CRISPR-Cas enzymes in microfluidic systems, CRISPR-on-chip platforms offer key advantages such as single-molecule sensitivity, multiplex detection, and applicability. However, integration with microfluidics for PoC applications is still poorly understood, despite CRISPR-Cas being widely used. This study reviews recent developments in CRISPR-on-chip-based diagnostics and highlights its potential applications in infectious diseases, biosensors, and personalized medicine. Furthermore, challenges and future perspectives in achieving an ideal diagnostic solution are discussed.

## Graphical abstract

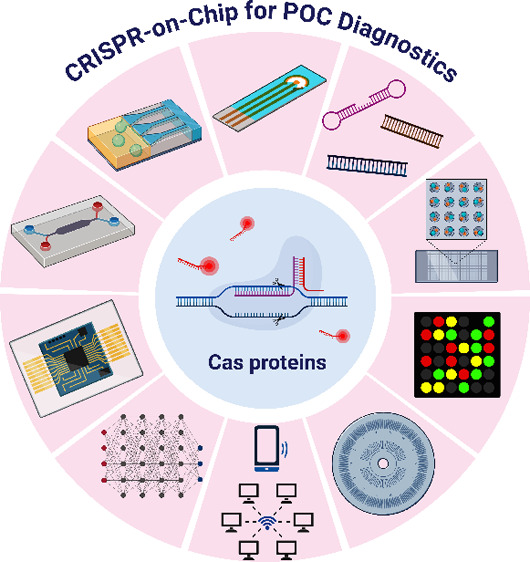

## 1 Introduction

Conventional molecular diagnostic platforms, such as PCR, qPCR, and next-generation sequencing (NGS), face significant challenges in achieving consistent applicability, particularly in regions with limited healthcare infrastructure.^1–6^ These challenges stem from their high costs, requirements for sophisticated equipment, and dependence on highly skilled personnel.^7^ In this context, clustered regularly interspaced palindromic repeats (CRISPR)/CRISPR-associated protein (Cas) systems, which had initially been utilized for gene editing purposes, have become effective diagnostic tools owing to their high specificity, sensitivity, and seamless integration capability with diverse platforms.^8–13^ The unique properties of Cas effectors make CRISPR-based platforms provide quick, affordable, and portable substitutes.^8,12,14,15^ Furthermore, obstacles, e.g., clinical validation, signal amplification needs, and limit of detection, continue to prevent widespread adoption.^7,12,13,16^

Microfluidic systems have become a game-changer in diagnostics, allowing for highly sensitive, miniaturized, and high-throughput detection.^16,17^ These systems are ideal for point-of-care (PoC) applications because they facilitate quick, automated, and cost-effective disease detection by combining numerous laboratory functions onto a single chip.^12,18–29^ Technological developments in fabrication, employing materials like polydimethylsiloxane (PDMS), glass, and other polymers, have produced adaptable microfluidic platforms that integrate multiple detection techniques, such as optical and electrochemical methods.^30,31^ Advances in microfabrication and 3D bioprinting have enabled faster, more complex, and cost-effective chip designs.^32,33^ By combining advanced fluid control mechanisms and nucleic acid extraction systems, developments in microfluidic technologies have produced fully automated molecular diagnostics.^12,19,34^ Molecular diagnostic capabilities have been greatly enhanced by developments in the microfluidics area, especially in precision medicine and the detection of infectious diseases.^16^

CRISPR-on-chip, the combination of CRISPR-Cas systems and microfluidic technology, is a major advancement in diagnostics.^15,17,35^ CRISPR-on-chip allows for ultrasensitive, real-time, and portable diagnostic solutions by fusing the automation, miniaturization, and quick processing capabilities of microfluidic platforms with the programmability and high specificity of CRISPR-based detection.^17,19,36^ With single-molecule sensitivity, these systems have shown great promise in identifying genetic mutations, infectious diseases, and cancer biomarkers.^37,38^ Combining microfluidic channels, stimulation elements, embedded biosensors, and finely tunable microenvironments can be developed.^32,33^ Using modern bioprinting techniques vascularized and viable tissue models with functional maturity can now be fabricated. These models can be patient-derived, leading to higher personalization and precision. With the use of microfluidics in patient-derived on-chip models, individualized drug screening and disease modeling can be achieved. CRISPR-integration can further expand the capabilities of such platforms, paving the way for precision diagnostics and personalized testing on standardized miniaturized models.

CRISPR-based diagnostics are now even more accessible and effective in accordance with developments in microfluidic architectures, such as polymer-based, centrifugal, paper-based, and digital microfluidics.^12,37,39–43^ The seamless integration of Deep Learning (DL), Artificial Intelligence (AI), and Internet of Things (IoT) will enhance PoC usability, automate result interpretation, and optimize data analysis.^44–47^ This review explores the integration of CRISPR-Cas platforms with microfluidic chip systems, collectively referred to as CRISPR-on-Chip, and highlights recent breakthroughs and emerging applications that demonstrate their transformative potential in molecular diagnostics and personalized medicine.

## 2 CRISPR-Cas System: Evolution, Mechanism, and Diagnostic Potential

The CRISPR/Cas system is essentially defined as an immune system mechanism improved by prokaryotic organisms throughout evolution against bacteriophage infections.^48,49^ The engineered CRISPR/Cas system provides accurate detection via specific activation of Cas proteins led by the gRNA that is uniquely designed to target foreign genetic material.^6,11^ These modified CRISPR/Cas systems have extensive applications in fields such as gene therapy in medicine, cancer, drug development, tissue engineering, and industrial/agricultural biotechnology.^50,51^ CRISPR-Cas platforms have recently evolved into a powerful diagnostic tool, leading to promising innovative applications.^52^

CRISPR sequences were first discovered by Ishino et al. in the *Escherichia coli* genome.^53^ Five unusual repetitive sequences were identified while sequencing of the *iap* gene, which is responsible for alkaline phosphatase isozyme conversion. The biological function of these repetitive sequences in the genome remained unclear and was classified as “cryptic sequences” for years. Research conducted by Dr. Mojica and colleagues in the 1990s began to shed light on the mystery surrounding CRISPR sequences.^54,55^ Similar repetitive sequences were found in the archaeal species *Haloferax mediterranei*.^54,56^ In 1996, these sequences were also identified in cyanobacteria and were termed long tandemly repeated repetitive (LTRR) sequences.^57^ Three independent research groups revealed in the early 2000s that CRISPR sequences contain foreign genomic sequences originating from bacteriophages and plasmids.^55^ This breakthrough advanced the idea that CRISPR is a unique part of the prokaryotic adaptive immunological system.^56,58,59^ In these findings, it has been stated that target genetic material is cleaved through Cas proteins’ activity based on immunological memory, and the mechanism has particularly evolved against bacteriophage infection.^11,49^ In 2012, Emmanuelle Charpentier and Jennifer A. Doudna introduced CRISPR-Cas9 as a genome-editing tool.^60^ Their research demonstrated that the Cas9 endonuclease could perform precise DNA cutting under the guidance of a programmed single-guide RNA (sgRNA).^49,60^ Due to its revolutionary impact on genetic engineering, this discovery was awarded the 2020 Nobel Prize in Chemistry.^61^

CRISPR-Cas can be classified under two main divisions (classes I and II) and various subtypes (Type I, III, IV/Type II, V, VI).^49,62^ CRISPR sequences are present in approximately 40 and 90% of bacterial and archaeal genomes, respectively, and definitive evidence for their presence in eukaryotic organisms has not yet been obtained.^55^ Ninety percent of CRISPR-Cas systems are class I systems, which are made up of several protein domains with distinct roles.^62^ Class II systems, on the other hand, are considerably less prevalent in nature and function with a single protein subunit. Generally, CRISPR systems used in diagnostics are divided into five main categories: Cas9, Cas12, Cas13, Cas14, and Cas10. In gene editing and therapeutic studies where high sensitivity and sequence compatibility are strictly considered, the Cas9 enzyme (Tip II) shows specific cleavage activity against target DNA.^11,63^ The Cas10 system recognizes RNA targets and initiates the cleavage activity after precise pairing; this system belongs to type III and has been effective in SARS-CoV-2 diagnosis.^64^ Particularly, Cas12 (Tip V) and Cas13 (Tip VI) systems have been widely used in diagnostic platforms.^6,65^ Cas12 and Cas13 systems exhibit collateral cleavage activity upon target recognition, with Cas12 targeting double-stranded DNA (dsDNA) and triggering nonspecific single-stranded DNA (ssDNA) cleavage.^63^ In contrast, Cas13 targets ssRNA and induces collateral ssRNA cleavage.^63^ In CRISPR-based diagnostic platforms, Cas12 exhibits trans-DNase activity depending on the recognition of the DNA target, while Cas13 exhibits trans-RNase activity following the recognition of RNA targets.^66^ Unlike the traditional approach, recent studies have revealed that Cas12 and Cas13 enzymes might display different recognition and cleavage activities.^66^ It has been reported that Cas12a can, under certain conditions, recognize not only DNA but also RNA targets under certain conditions and exhibit trans-RNase activity in this process.^67^ In addition, Cas13a, known as an RNA-specific nuclease, has been suggested to recognize DNA targets and exhibit trans-cleavage activity with high specificity.^68^ These findings may expand the versatility and application potential of CRISPR-Cas enzymes in diagnostics, contributing to developing sensitive, specific, more flexible and robust platforms for detecting both DNA and RNA-based pathogens.

Despite its broad range of potential applications, CRISPR/Cas systems currently have some limitations. Off-target editing has remained one of the primary concerns regarding Cas protein activity. While several methods have decreased off-target activity and optimization studies have shown great potential, a consensus method has not been reached yet.^69^ While CRISPR-based diagnostics have demonstrated high specificity and target recognition, achieving reliable and robust single-base specificity has remained challenging.^70^ Sample preparation and extraction steps have introduced the risk of contamination, leading researchers to explore CRISPR-integrated nucleic acid amplification methods and single-pot assay formats.^6,65,71^ Quantification has remained limited with a majority of CRISPR assays producing qualitative or semiquantitative outputs.^44^ Multiplexed assays have been developed, however, throughput trade-offs have limited scaling. Some panels and more complex designs have had to compromise on sensitivity/specificity or required cold chains, limiting use in resource-limited settings.

While limitations have not been completely addressed, significant progress has been achieved, allowing the field of CRISPR-based diagnostics to gain widespread attention. In CRISPR-Cas-based detection mechanisms, signal amplification and detection steps are carried out using various techniques after nonspecific cutting activity dependent on the target sequence (Figure 1). In this context, fluorescence-based reporting systems, lateral flow assays, and electrochemical sensors are widely used in CRISPR-based diagnostic platforms. In particular, electrochemical detection strategies offer significant advantages in real-time, automatic, and quantitative diagnostic processes by showing high compatibility with microfluidic-based systems, and digital diagnostic platforms.^72^ In addition, using reporter molecules with different properties increases the sensitivity of the relevant detection methods. It facilitates the integration of platforms into PoC applications.^73^ The use of colorimetric assays, biotin-based labeling systems, turbidity-based detection, gold nanoparticle-based sensors, and polyelectrolytes play a critical role in increasing the specificity and sensitivity of CRISPR-based diagnostic systems.^73^ The new-generation CRISPR-on-Chip technology offers low-cost, high-sensitivity, and specific diagnostic capabilities while also enabling field applications thanks to its minimal laboratory equipment requirements.^74^ Additionally, the integration of CRISPR-on-Chip systems with AI-supported analyses, wearable biosensors, and mobile phone-based detection systems has become one of the main reasons for the increasing interest in this technology in the field of diagnostics in recent years.^75,76^

**Figure 1:**
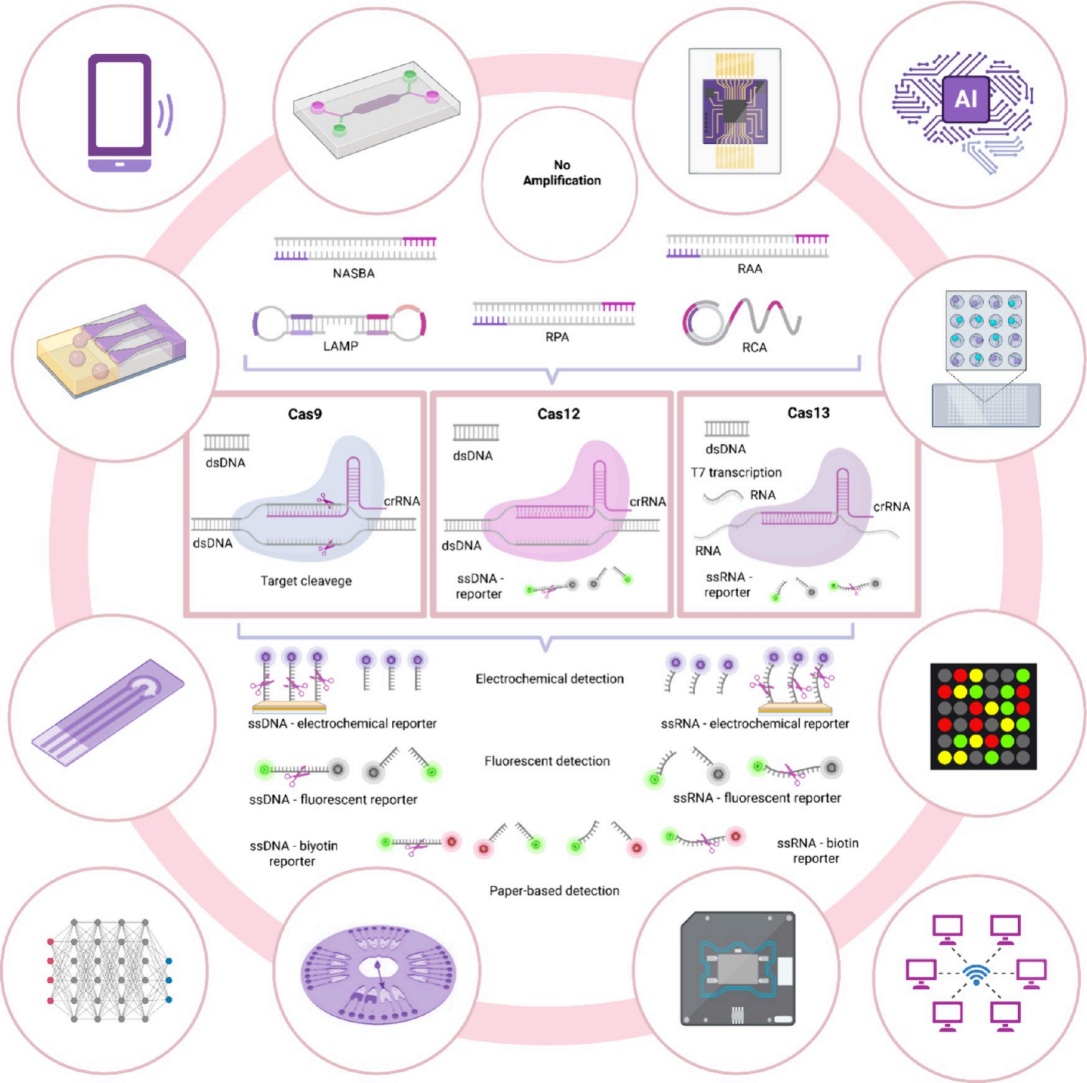
CRISPR-on-Chip technologies are illustrated with various schematics. CRISPR detection technology can be amplification-free or combined with a preamplification step. Nucleic acid amplification techniques such as NASBA, RCA, RAA, LAMP, and RPA are used for preamplification. Cas9, Cas12, and Cas13 are the most used enzymes in diagnostic systems. While Cas9 has a specific target cleavage effect, Cas12 and Cas13 have a nontarget cleavage effect. Cas9, Cas12, and Cas13 cleave dsDNA, ssDNA, and ssRNA, respectively. The endonuclease activities of Cas enzymes allow the use of reporters. These reporters serve for fluorescence detection, colorimetric detection (lateral flow strip/paper-based), and electrochemical detection. CRISPR technology can be combined with a wide variety of microfluidic systems. The most combined microfluidic systems include polymer-based, paper-based, droplet-based, digital, and centrifugal types. With support for smartphones, ML, and AI, it enables high-accuracy results collection and data comparison.

## 3 CRISPR-Cas-Based Diagnostic Modalities

### 3.1 Nucleic Acid–Based Platforms

Nucleic acid–based diagnostic methods can be examined in two categories: preamplification-based and amplification-free systems. Thanks to highly efficient amplification tests, optimum sensitivity is observed especially in the diagnosis of acute and chronic diseases caused by infectious diseases.^73^ Polymerase chain reaction (PCR), recognized as the gold standard among amplification tests, offers significant advantages, including high sensitivity and specificity.^77^ However, it also presents notable drawbacks, such as the requirement for sophisticated equipment, trained personnel, and high operational costs.^73,78^ In addition to PCR, some isothermal nucleic acid amplification tests have also been developed and applied in various CRISPR-Cas based diagnostic platforms.^77^ In another field, in the platforms that do not include upstream amplification, samples with high analyte concentrations can be used for targets such as microbial genome, human genomic DNA, human mRNA, and miRNA, and the detection limit is usually observed at the picomolar level.^73^

In the field of point-of-care diagnosis, the goal is to create user-friendly, fast, and advanced equipment-free platforms.^6,78^ Considering these criteria, the development and improvement of platforms that do not require preamplification are important for the field of diagnosis. However, more research is needed to increase the sensitivity of preamplification-free CRISPR-Cas-based diagnostic platforms so that they can be used as a reliable method for clinical needs.^73^ To overcome these limitations, various approaches such as multiple crRNA-based determination, signal cascade amplification, and the use of digital technologies have been investigated.^79^

#### 3.1.1 Amplification-Based Platforms

Two-step CRISPR-Cas-based diagnostic systems rely on the principle that the target nucleic acid is first amplified using a PCR or an isothermal amplification method, and then a specific readout is obtained through CRISPR-Cas activity in another environment.^6,65,79^ In these methods, isothermal techniques such as recombinase polymerase amplification (RPA), loop-mediated isothermal amplification (LAMP), and nucleic acid sequence-based amplification (NASBA) are used to obtain high amounts of target nucleic acid in a short time.^6,79^ For example, the allele-specific sensing ability of Cas13a has been combined with RPA to detect pathogens at attomolar levels.^80^ While these two-step platforms offer high sensitivity and specificity, they may also have disadvantages such as processing time and risk of contamination.^71,81^ Pardee et al. developed a platform (NASBACC) that combines NASBA isothermal amplification and CRISPR-Cas9-based toehold switch reactions for the detection of Zika virus.^27^ This paper-based, low-cost, and portable platform provided specific detection of Zika virus strains with fM-level sensitivity and became one of the leading studies of the period in the field of CRISPR-Diagnostics (CRISPR-Dx).^27^

In CRISPR-Cas-based diagnostic platforms, amplification of the target nucleic acid is of critical importance for situations requiring ultrasensitivity. In addition to the two-stage detection involving CRISPR-Cas activity after preamplification, “one-pot” platforms that combine both processes in a single stage have also been developed. In the face of possible contamination risks that may negatively affect the performance of the system in two-stage processes, one-pot systems have recently gained attention especially in the field of PoC diagnostics.^82^ In a study conducted by Zhou et al., the CRISPR-one (CRISPR-mediated testing in one tube, one pot) platform, dependent on CRISPR-AapCas12b activity, was developed based on multiple cross-displacement amplification of the DNA segment encoding the community-acquired respiratory distress syndrome toxin of *M. pneumoniae*.^83^ This platform, which shows significant sensitivity compared to its microfluidic chip and real-time PCR counterparts and operates in a single reaction environment, has been described as promising in clinical studies due to the specificity, convenience, and speed it provides.^83^

#### 3.1.2 Amplification-Free Platforms

Currently, molecular diagnostic technologies have advanced markedly in specificity and sensitivity, with novel CRISPR-based platforms providing innovative solutions in this domain. Detection of biomarkers at low concentrations has become possible, especially thanks to selective targeting of nucleic acids and signal generation techniques.^38^ DASH (Depletion of Abundant Sequences by Hybridization) targets unwanted nucleic acid sequences at high concentrations via Cas9, providing sensitive detection of target sequences at trace amounts, especially in metagenomic pathogen analyses.^84^ Rather than being a diagnostic platform, this system is optimized to increase detection sensitivity in existing platforms (e.g., NGS applications) and in areas such as cancer detection (cancer profiling).^84^ The acCRISPR-PTS-SERS platform is an innovative method for detecting hepatocellular carcinoma biomarkers. It combines surface-enhanced Raman spectroscopy (SERS) with CRISPR-Cas technologies on pregnancy test strips, marking a significant advancement in biomarker detection.^85^ In this system, CRISPR-Cas technology was used for signal amplification, overcoming the low sensitivity and limitations in quantitative analysis.^85^ In addition, the use of gold nanoparticle-based core–shell structured Raman nanoprobes increased the detection sensitivity to the femtomolar level, allowing ultralow detection limits to be achieved.^85^ The method offers high specificity, accuracy, and stability in serum samples and is considered a prospective strategy for automated and quantitative analysis by integrating with microfluidic chips and smartphone technology.^85^ DC-ECL-CRISPR emerged as a sensitive RNA detection method that does not require RNA amplification.^86^ This method is capable of detecting *E. coli* RNA with high specificity. The platform provides two-step signal amplification using Ru­(II)-PLL complex and CRISPR/Cas13a.^86^ In particular, the integration of dry reagents into the lateral flow chip eliminated additional processing steps and reduced the total detection time to 20 min.^86^ The effective implementation of this approach in clinical samples and its versatility for biomarker and heavy metal studies suggest substantial potential for incorporation into extensive diagnostic platforms.^86^

### 3.2 Non-Nucleic Acid–Based Platforms

CRISPR-based diagnostic methods can also be used to detect non-nucleic acid biomarkers such as small organic molecules, proteins, and metal ions.^87^ In these applications, CRISPR acts as a signal enhancer or reporting tool, while detection of the target molecule is achieved through conformational changes in proteins or aptamers.^73^ In addition, new approaches such as allosteric transcription factors (aTFs) and CRISPR-responsive hydrogels offer great potential in PoC diagnostic systems, while the specificity and applicability of these platforms have increased with the use of functional DNA (fDNA).^87^ The CRISPR-based Ultrasensitive Immunoassay (CRUISE) technology, recently developed, integrates CRISPR-Cas12a enzymatic activity with conventional immunoassay techniques, facilitating the highly sensitive identification of non-nucleic acid targets.^88^ CRUISE optimizes the target recognition process by using antibodies conjugated with single-stranded DNA (ssDNA) instead of aptamers or allosteric transcription factors (aTFs) and increases signal amplification by binding more than one ssDNA per antibody. This platform, which shows 1000 times higher sensitivity compared to traditional ELISA tests, has brought an innovative approach integrated with traditional immunoassay methods.^88^

## 4 CRISPR-Cas and Microfluidics: Next-Generation Diagnostic Platforms

### 4.1 Sample Preparation Strategies and Their Integration into Microfluidic Systems

For CRISPR-based diagnostics to be effectively utilized in PoC settings, it is critical to underline the upstream processing steps. These include sample preparation, nucleic acid extraction, amplification, transduction, target recognition, and detection. All processing steps significantly impact the overall sensitivity, specificity, and usability of diagnostic systems in resource-limited environments.^89^ Microfluidic systems have constraints in portability owing to their reliance on external instruments such as pumps and power supplies.^90^ Instead, these devices can be substituted with integrated, smaller components, increasing their scalability and reliability in point-of-care settings.^90^ From this point of view, sample preparation, purification, and concentration of target analytes are essential requirements for developing CRISPR-based microfluidic systems suitable for field use.^79^ Pretreatment of clinical samples can be done with more straightforward methods, such as filtration that does not require centrifugation on microfluidic platforms, and cell disruption can be integrated with thermal, chemical, enzymatic, mechanical, or electrical methods.^91^ Nucleic acid extraction can be performed by liquid–solid, solid–solid, and magnetic bead extraction methods. In addition, the isotachophoresis method, which is compatible with amplification methods and CRISPR-Cas reaction and can be integrated into microfluidic systems, has recently attracted attention.^92^

### 4.2 Microfluidic CRISPR-Dx Platforms

Diagnostic capabilities and practical usability of CRISPR-Cas systems can be improved substantially by leveraging micro- and potentially nanotechnologies.^93^ Microfluidics can help streamline automation, improve sensitivity, and enable multiplexing. Successful CRISPR PoC devices couple sample-to-answer workflows within closed microfluidics systems to minimize contamination and user dependency.^89^ Steps such as on-chip extraction or amplification directly from clinical samples can be integrated depending on platform goals.^6^ Then, a detection modality is selected. Electrochemical and optical/fluorescent detection systems are widely adopted. The detection modality can enable amplification-free diagnostics, quantification, and multianalyte assays.^73^ Performance optimization can be achieved using a wide range of strategies including gRNA design optimization, chip-surface chemistry modification, or reaction environment (i.e.) alteration.^94^ Final steps for clinical and PoC translation include multiplexing, balancing sensitivity, cost, and robustness, data integration, or adopting user-friendly elements.^95^

#### 4.2.1 Polymer-Based CRISPR-Microfluidic Chips

Polymer-based microfluidic systems offer innovative diagnostic platforms for the rapid and sensitive detection of nucleic acids when integrated with CRISPR systems. Polymers such as polydimethylsiloxane (PDMS) and poly­(methyl methacrylate) (PMMA) are frequently preferred in the development of such integrated systems due to their low-cost production, optical transparency, and biocompatibility.^90,96^ Other materials can be thermoplastics (e.g., polystyrene, polycarbonate, and cyclic olefin copolymer), glass, and silicon.^96^ Techniques such as hot embossing, laser cutting, and 3D printing are frequently employed in the production of these devices.^90,96^

Ren et al. developed a one-pot PDMS/Glass chip platform based on portable RPA preamplification and CRISPR-Cas12a trans-cleavage activity that can directly detect nucleic acids of intestinal pathogens (Figure 2a).^39^ The essential features of the platform are its ability to detect multiple samples—*E. coli*, *Pseudomonas aeruginosa*, and methicillin-resistant *Staphylococcus aureus*—on a single chip, its high programmability, and its usability for the detection of other targets by changing the crRNA and RPA primers.^39^ A PDMS chip system for the detection of SARS-CoV-2 with CRISPR/Cas13a was able to diagnose with a sensitivity of 100 copies/μL in just 30 min using mobile phone microscopy^97^ The IMPACT (Integrated Micropillar Polydimethylsiloxane Accurate CRISPR Detection) system offers Cas12a-mediated viral DNA detection on a PDMS-based microfluidic system.^98^ Chip surfaces designed with a micropillar structure are coated with streptavidin to immobilize ssDNA probes. Fluorescent signals are generated in the presence of target DNA due to the collateral cleavage activity of Cas12a. This design enables highly sensitive detection in the 0.1–1 nM range.^98^ The CASCADE system was able to detect SARS-CoV-2 RNA without CRISPR/Cas12a-based amplification on a PMMA-based microfluidic chip.^99^ Gas bubbles generated by catalase-bound ssDNA probes are read with a mobile phone camera, providing a highly sensitive (∼50 copies/μL) and portable diagnostic platform.^99^

**Figure 2:**
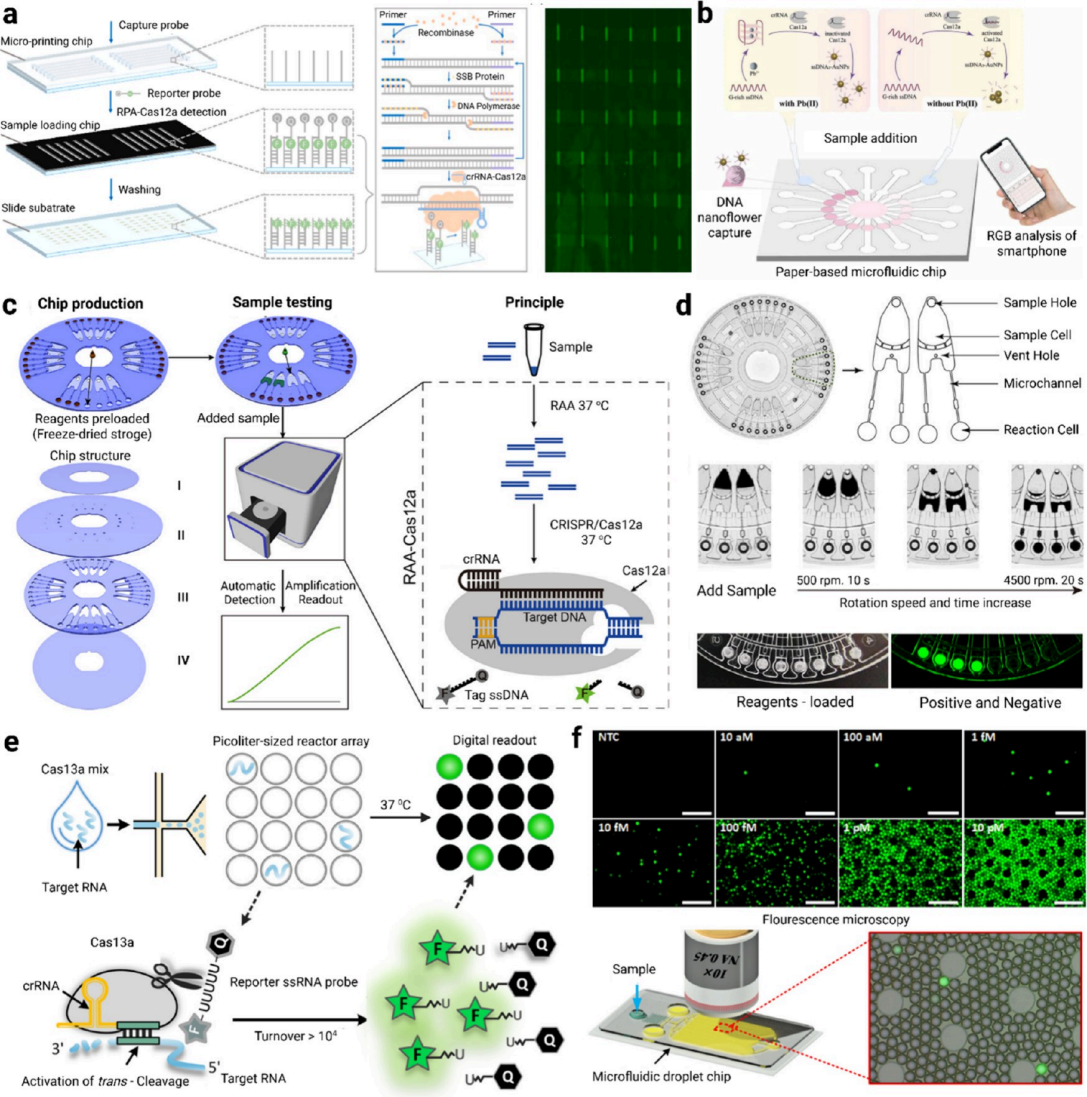
(a) The integration of a polymer-based one-pot chip for the RPA-CRISPR/Cas12a reaction is schematized. The RPA-CRISPR/Cas12a one-pot reaction principle and the on-chip reaction results on a microarray fluorescence scanner are demonstrated. Adapted with permission from ref 39. Copyright 2024 Elsevier Inc. (b) The operating principle of a paper-based microfluidic biosensor that detects Pb­(II) using a CRISPR-Cas12a-enabled, smartphone-based device is illustrated. The presence of Pb­(II) inhibits Cas12a activation via G-quadruplex structures, preventing the dispersion of gold nanoparticles. The dispersed nanoparticles are captured by DNA nanoflowers in the detection region, resulting in a color change. Adapted with permission from ref 40. Copyright 2022 Elsevier Inc. (c) RAA and CRISPR-Cas12a reactions are integrated and automatically carried out on a PMMA-based disc-shaped centrifuge microfluidic (lab-on-a-disc) system. Adapted with permission from ref 41. Copyright 2020 American Chemical Society. (d) The structure of the lab-on-a-disc and the resulting fluorescent signal are detected by readout in approximately 1.5 h. Adapted with permission from ref 41. Copyright 2020 American Chemical Society. (e) The operating principle of Cas13a-based RNA detection on the droplet microfluidic platform is presented. By isolating target RNA molecules in each droplet, the RNA-triggered catalytic activity of the Cas13a enzyme is enhanced, enabling specific and amplification-free RNA detection. Adapted with permission from ref 12. Copyright 2021 American Chemical Society. (f) Digital RNA quantification is achieved at the single-molecule level by measuring signal intensity within the droplets using fluorescence microscopy on the droplet microfluidic chip. Adapted with permission from ref 12. Copyright 2021 American Chemical Society.

#### 4.2.2 Paper-Based CRISPR-Microfluidic Chips

Low-cost and manufacturable microfluidic platforms have garnered significant interest in recent years thanks to their ease of fabrication and environmental compatibility. In particular, paper-based microfluidic devices are easy to fabricate by arranging hydrophobic and hydrophilic areas to regulate fluid flow, and they can be safely disposed.^90^

Xu et al. introduced a portable paper-based biosensor for the detection of miRNA-141 based on CRISPR-Cas12a-Glucose oxidase (GOD) cleavage and peroxidase (POD)-like catalytic activity of AuPtPd@GOD nanozyme imprinted on graphdiyne oxide (GDY).^100^ In this study, ultrasensitive and real-time analysis at the aM level was carried out by colorimetric and electrochemical dual-detection methods. The sensor tested in human serum samples transmitted to a smartphone via Bluetooth; color change due to oxidation of the TMB indicator by AuPtPd@GDY was assessed by camera and application on the smartphone.^100^ This innovative platform introduces a promising approach for PoC tumor biomarker detection.^100^ In a study, it was designed by a paper-based microbiosensor chip where the CRISPR-CAS12a technique is applied and PB­(II) ions were made in place, low-cost and rapid detection (Figure 2b).^40^ The sensor produces visual signal based on color change by suppressing CAS12a activation through Pb­(II)-induced G-quadruplex structure. Quantitative analysis can be done with the smartphone application. The method is compliant with high specificity, determination, and field applications.

PLACID (Paper-based LAMP-CRISPR Integrated Diagnostics) platform is a low-cost, biocompatible, portable, and sensitive molecular diagnostic tool that integrates LAMP and CRISPR-Cas12a on a paper-based chip.^101^ Sample processing, heating, and fluorescence-based detection are performed on a single chip under the monitoring of Arduino UNO R3 microcontroller. PLACID provides optimum detection through colorimetric/luminance analysis supported by a Bluetooth-connected smartphone application. It offers significant potential for field applications with its stability for up to 8 weeks at room temperature.^101^

#### 4.2.3 Digital CRISPR-Microfluidic Chips

Lab-on-a-Chip (LoC) technologies can be precisely and flexibly manipulated using digital microfluidics, which allows for the controlled manipulation of discrete droplets, usually in the nanoliter to microliter range.^102^ Digital CRISPR-based microfluidic systems provide highly sensitive and quantitative detection by dividing diagnostic reagents into multiple microreactors (droplets or microchambers). These systems are typically constructed with droplet microfluidics or microchamber arrays and can optimize local environments, enhancing CRISPR enzyme activity.^103^ Digital microfluidics has the major benefit of not requiring traditional external instruments, like pumps and valves, which are typically necessary for continuous-flow microfluidic systems.^102^ These CRISPR-integrated platforms use fewer reagents than traditional methods, are amenable to automation, and offer single-molecule analysis (Figure 2e,f).^12^

Tian et al. used a droplet-based CRISPR platform to detect 16S rRNA, microRNA, and SARS-CoV-2 at the single-molecule level.^12^ Systems such as RADICA^43^ and deCOViD^104^ have eliminated the need for a PCR thermocycler, enabling detection through isothermal amplification. However, deCOViD can cause overamplification of targets at low temperatures.^104,105^ To address this issue, Ding et al. used a warm-start CRISPR method developed by MC-DRP that combines DAMP/RT-DAMP and Cas12a to detect 5 copies/μL of SARS-CoV-2 RNA in 90 min.^106^

An RPA-CRISPR-based digital microfluidic platform was designed for *Helicobacter pylori* virulence genotyping and detection by Liu et al.^107^ The platform is capable of multitarget analysis by simultaneously detecting cagA, vacA, and ureB genes. This digital CRISPR-based integrated chip system offers rapid and highly sensitive diagnosis by reducing the reaction time to 30 min. However, chip-based nucleic acid extraction’s lower efficiency than traditional methods has been stated as an important limitation of the system.^107^ For multipathogen detection, the CARMEN v.1 system was able to simultaneously detect 169 viruses using color coding and droplet matching (Figure 3).^37^ In this study, an mChip architecture designed to scale the droplet-based workflow was also introduced, increasing the number of microwells to approximately 4500 and thereby substantially enhancing the overall testing capacity of the system (Figure 4a–g).^37^ However, the system’s specialized hardware requirements and 8–10 h workflow are limiting factors.^37^ These limitations arise due to the droplet generation process, the color-coding process, and the need for specialized imaging setups. Therefore, the developed mCARMEN platform can detect 21 viruses and distinguish SARS-CoV-2 variants with nearly 100% accuracy.^15^ The IFC-based approach eliminates the need for droplet handling in the mCARMEN platform, accelerating the workflow by enabling the automatic on-chip mixing of samples and detection reactions within 96 × 96 or 192 × 24 microfluidic cartridge formats (Figure 4h).^15^ Finally, Multiplexed Intermixed CRISPR Droplets (MIC-Drop) technology has demonstrated the feasibility of using digital microfluidics for in vivo CRISPR screening.^108^ The integration of 3D printing technology makes these systems more portable and sensitive.^42,109^

**Figure 3:**
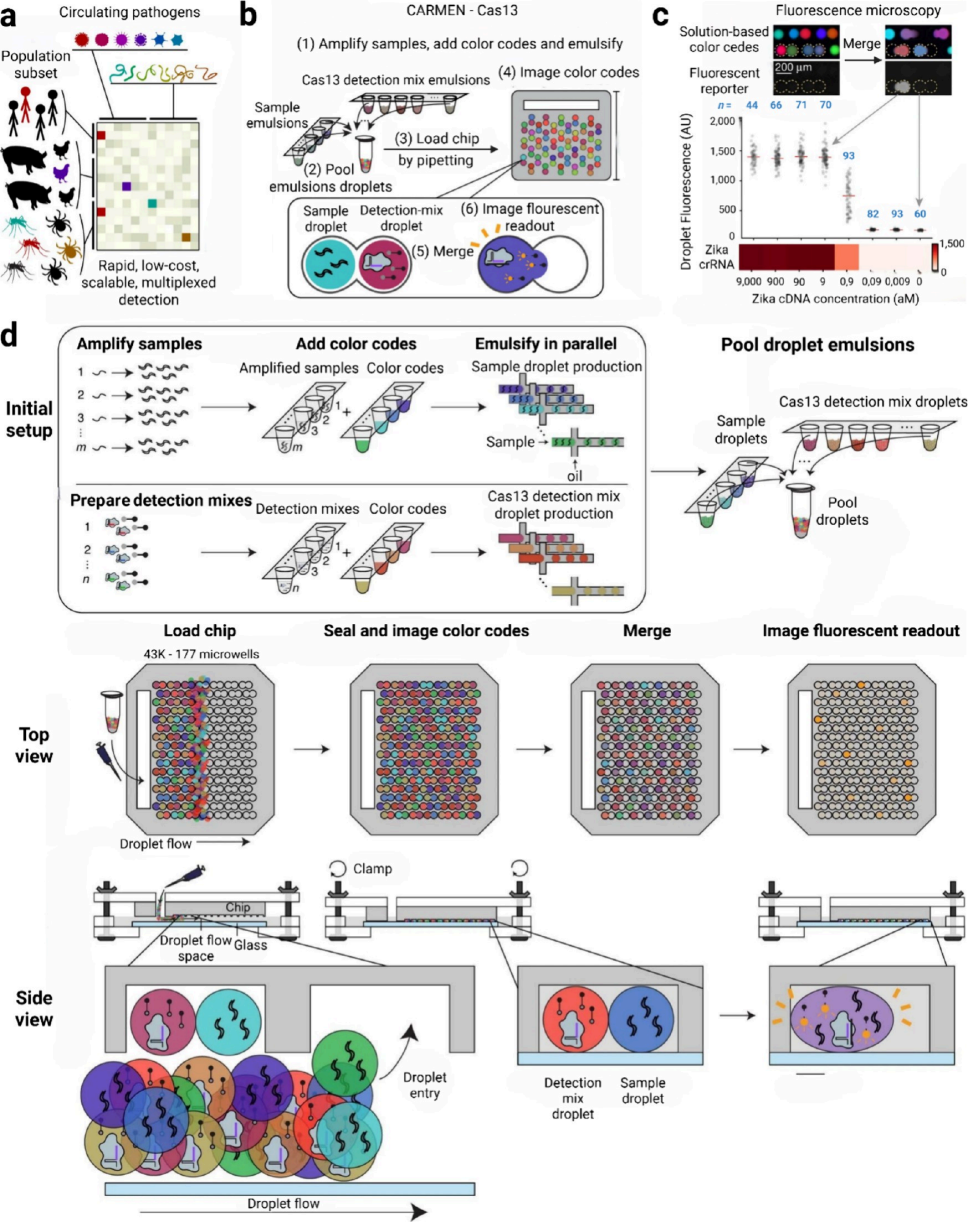
The CARMEN-multidroplet detection system is illustrated. Adapted from ref 37 under CCBY 4.0 license. Copyright 2020 Springer Nature. (a) Need for multiplex detection of circulating pathogens in human and animal populations. (b) The principle of CARMEN-Cas13 is schematized. (c) Droplet fluorescence readout results for Zika virus detection. (d) Stages of the multidroplet microfluidic system; preparation of samples, creation of pool droplet emulsions, loading into the microfluidic system, and droplet flow progression are presented.

**Figure 4:**
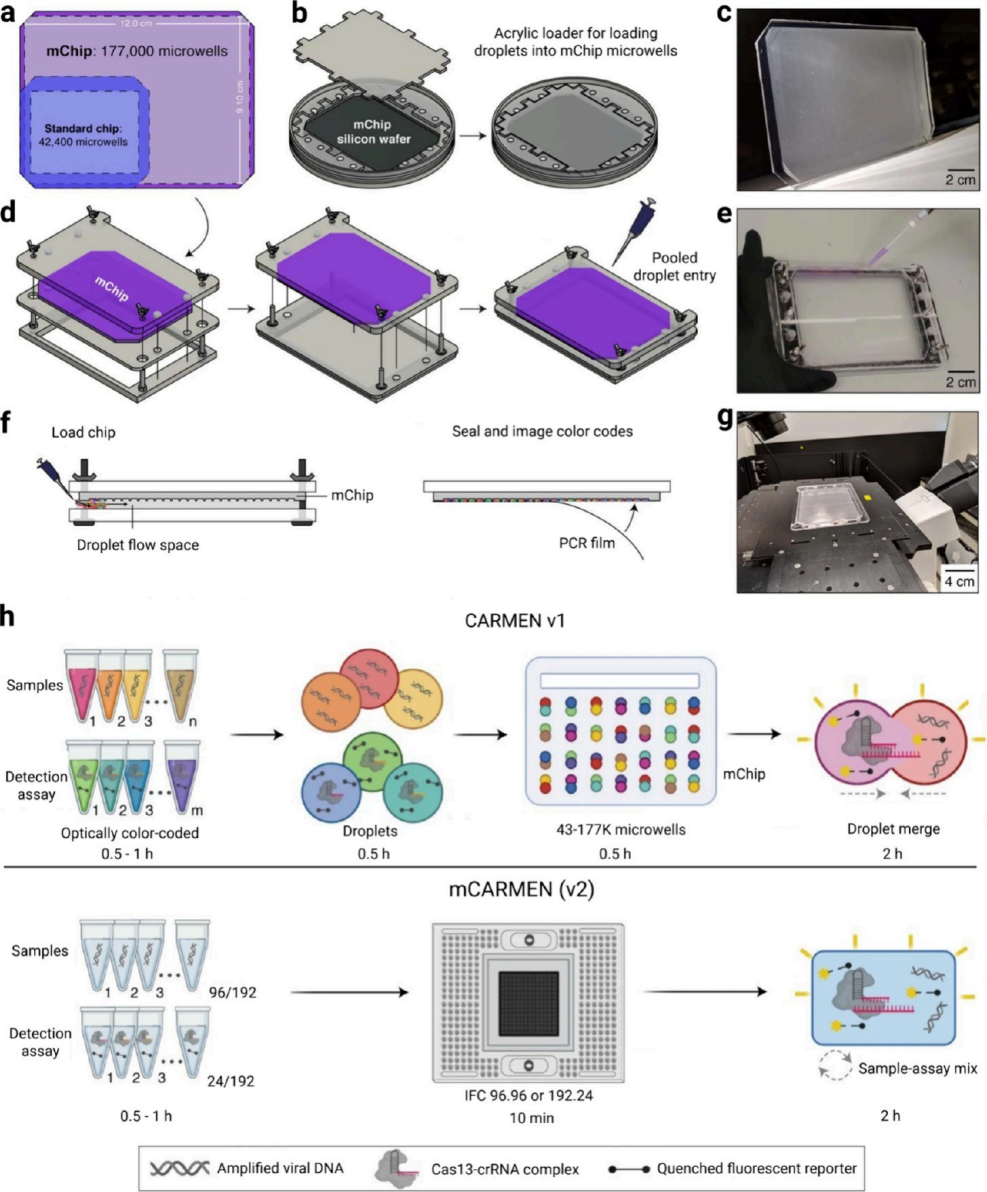
Overview of the mChip architecture (a–g) (adapted from ref 37 under CCBY 4.0 license. Copyright 2020 Springer Nature.) and the IFC-based mCARMEN workflow (h) (adapted from ref 15 under CCBY 4.0 license. Copyright 2024 Springer Nature). (a) Comparison of standard CARMEN v1. chip and the expanded mChip layout. (b) CAD drawings of the acrylic molds used to fabricate the high-density microwell array. (c) Photograph of the fabricated mChip showing the enlarged microwell region. (d) Modular loading system used to position the mChip during the filling process. (e) Introduction of the droplet pool onto the chip surface for microwell loading. (f) Workflow steps illustrating droplet settlement into microwells and sealing of the mChip. (g) Final configuration of the sealed mChip prepared for low-magnification fluorescence imaging. (h) Schematic representation comparing the mChip-based CARMEN v1 configuration with the IFC-enabled mCARMEN workflow, in which 96 × 96 or 192 × 24 microfluidic cartridges autonomously mix samples and detection reactions, eliminating droplet handling and accelerating the overall process.

#### 4.2.4 Centrifugal-Based CRISPR-Microfluidic Chips

Centrifugal microfluidics precisely controls fluid circulation inside microscale structures by using simple rotational forces.^110^ It is considered one of the most suitable platforms for rapid diagnostic systems, particularly PoC, due to its automation and integration.^110^

Peng et al. developed a centrifugal microfluidic array called Cas12-MRVDB (Multiple Respiratory Virus Detection Biosensor) based on isothermal RPA followed by CRISPR-Cas12a fluorescent detection.^111^ This chip was designed to simultaneously detect 14 viral RNAs, including MERS, SARS, and some clinical variants of SARS-CoV-2. This platform, which can operate at a relatively low temperature (39 °C), provided high specificity while maintaining detection accuracy and sensitivity comparable to fluorescent PCR methods and achieved this in a very short time of 30 min.^111^ However, the failure to detect some SARS-CoV-2 mutant Omicron strains and the relative difficulties in stable storage conditions of RPA-CRISPR reaction reagents have raised some questions about the sensitivity of the platform.^111^ Chen et al.’s CASMEAN system integrates RAA and CRISPR-Cas12a-based nucleic acid detection with a PMMA-based centrifugal microfluidic disk (Figure 2c,d).^41^ Within the disk, amplification and CRISPR reactions occur automatically in separate compartments. The Cas12a enzyme’s ability to cleave target DNA is utilized in a controlled manner to produce a specific fluorescent signal. As a result, the entire process can be completed in approximately 1.5 h, delivering outcomes with high sensitivity and specificity.^41^

The Automatic Microfluidic Diagnostic (AMIC) platform developed by Xiang et al. is a one-pot RAA-CRISPR/Cas13a-based system that utilizes a high-throughput fluorescent detection method.^112^ This system integrated the Chelex-100 chelating resin-based nucleic acid extraction method, which is comparable to the magnetic bead-based extraction method, into the microfluidic chip, reducing manual sample manipulation errors and shortening the total detection time to 40 min.^112^ However, the problems, such as neglecting sample enrichment and separation processes and the difficulty of detecting low-concentration bacteria, suggested that the system needs further optimization.^112^

Li et al. established the Lab-in-a-Magnetofluidic Tube (LIAMT) platform, which provides a portable, fully integrated molecular diagnostic system for nucleic acid–based viral diagnosis.^113^ The system combines viral digestion, nucleic acid extraction, isothermal amplification, and CRISPR detection processes using magnetofluidic micro/nano magnetic beads in a single microcentrifuge tube.^113^ With a sensitivity of 73.4 and 63.9 copies/mL, respectively, LIAMT was able to identify HIV and SARS-CoV-2. However, two of the platform’s shortcomings are the 1 h detection time and the need for precise sample preparation. By adding fully automated magnetofluidics and reagent lyophilization systems, the platform could become more portable and support its widespread use in resource-constrained areas.^113^

#### 4.2.5 AI- and Deep-Learning Integrated CRISPR-Microfluidic Platforms

DL- and AL-based analysis performed via fluorescence detection and visual outputs visible to the naked eye (readout) on CRISPR/Cas12 and CRISPR/Cas13 platforms offer innovative solutions for diagnostics.^114^ In this way, sensitive and reliable results can be obtained in quantitative analyses. A CRISPR/Cas12a-based microwell array PDMS chip has been developed to detect *Cryptococcus fungi* with DL algorithms (Figure 5f–h).^47^ This chip, which was designed as a portable and integrated smartphone diagnostic platform, has shown ultrasensitivity at the 0.5 pM level.^47^

**Figure 5:**
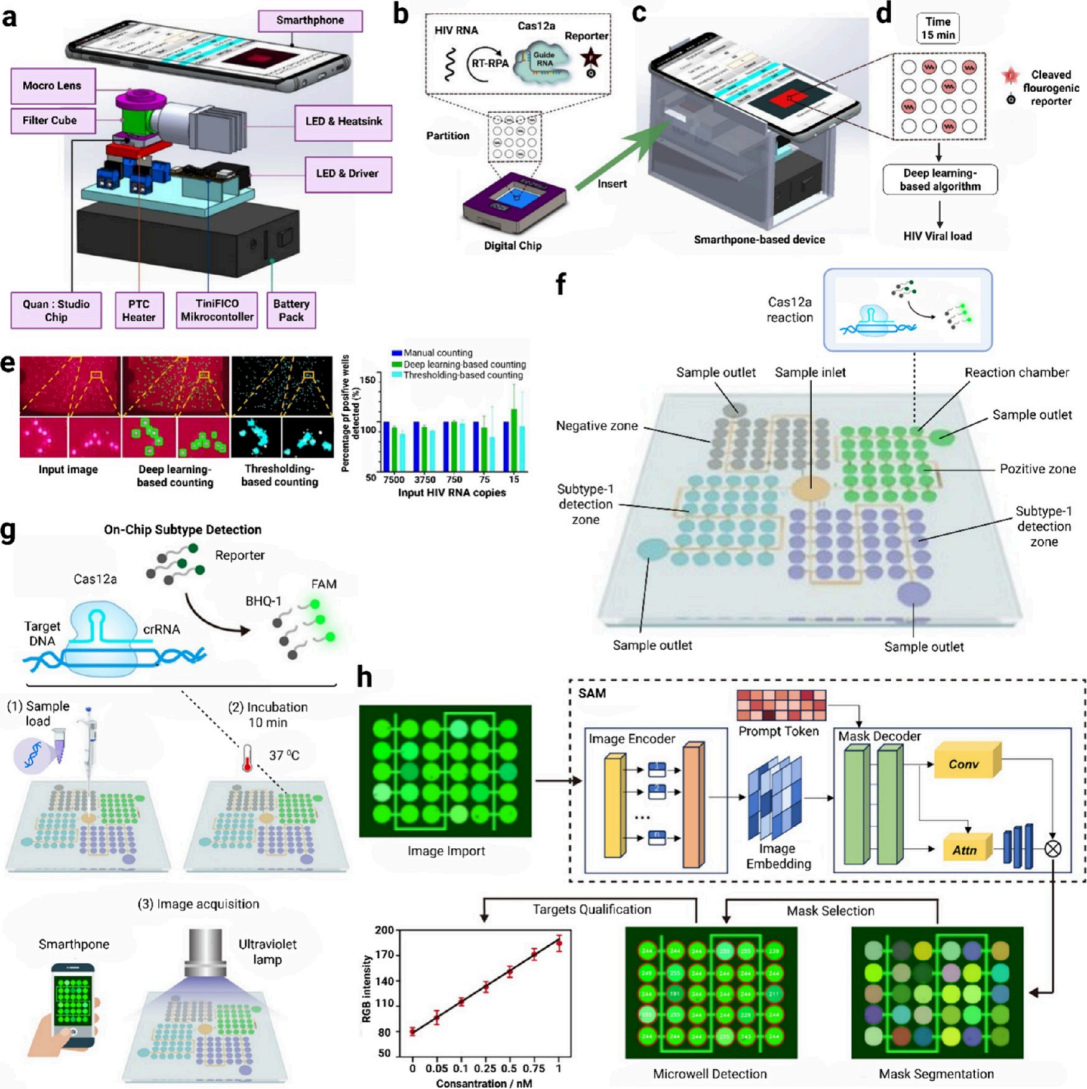
(a) Structure of the palm-sized device integrating thermal, optical, and smartphone modules. (b) RT-RPA-CRISPR assay on a digital chip. (c) Partitioned reactions on a digital chip running within a smartphone-integrated portable device. (d, e) Deep-learning-assisted fluorescence image analysis for accurate quantification of positive wells. (a–e) Adapted with permission from ref 46. Copyright 2023 medrXiv. (f) DEMA platform: architecture of the microwell array biological chip for rapid and sensitive detection of *Cryptococcus* subgroups via the CRISPR–Cas12a system. (g) Simplified schematic depiction of the testing steps performed on the chip. (h) Automatic recognition of well images and precise *Cryptococcus* enumeration using DL-assisted analysis. (f–h) Adapted from ref 47 under Creative Commons CCBY license. Copyright 2024 John Wiley and Sons.

A smartphone-compatible platform that integrates CRISPR-based diagnostic systems with digital microfluidic technology is one of the first systems reported for the rapid and quantitative detection of HIV RNA and offers a significant advance in local clinical applications (Figure 5a–e).^46^ This system, which performs advanced sample analysis with an improved DL algorithm based on YOLOv5, needs further optimization due to factors such as high cost and limited suitability for PoC applications.^46^ Zhang et al. constructed an innovative microfluidic platform called mutaSCAN by combining RT-LAMP and CRISPR-Cas12a with DL technology.^115^ This platform, which eliminates nucleic acid extraction, offers rapid, sensitive, and automated analysis while providing scalability and AL-supported real-time imaging.^115^ On the other hand, one of the critical shortcomings of the system that limits its use in PoC diagnostics is its dependence on some basic laboratory equipment.^115^

#### 4.2.6 Other CRISPR-Microfluidic Chip-Based Platforms

CRISPR-on-Chip platforms have evolved beyond traditional genetic diagnostics, enabling the detection of a broad range of targets, such as protein biomarkers, telomerase activity, and oncogenes. Various biosensing strategies and amplification techniques have been employed, and these systems are promising for future biomedical applications.^13^

Hu et al. exhibited a carbon nanotube-based field-effect transistor (CNT-FET) PoC testing platform, integrated with the CRISPR/Cas12a system.^116^ This platform enables ultrasensitive, specific, and real-time detection of cardiac troponin I (cTnI) protein.^116^ The platform cannot detect multiple biomarkers contained in whole blood samples, which is considered one of the platform’s main negative aspects.^116^ Also, the authors have concluded that further advancements in integrating microfluidics with the CNT-FET platform will be the upcoming goal.^116^ In this way, rapid isolation, enrichment, release, and simultaneous detection of different biomarkers associated with myocardial damage will be achieved.^116^

The CRISPR-Cas12a-based microfluidic platform reported by Jiang et al. was designed to detect telomerase activity at the single-cell level.^117^ The platform provides sensitive detection by signal amplification via UiO-66 nanoparticles, a metal–organic framework (MOF) carrying DNA strands that trigger CRISPR/Cas12a activation. However, complex sample preparation and difficulties encountered in cell isolation can be considered major shortcomings.^117^

Beyond pathogen diagnosis, CRISPR-on-Chip-based platforms offer significant potential in advanced biomedical applications, integrating with microfluidic chip technologies for genetic mutation detection.^118^ Many researchers in precision medicine (oncology) and cancer profiling have studied and evaluated this potential. A sensitive test was developed with a single crRNA using enzymatic recombinase amplification (ERA) combined with a modified CRISPR/Cas12a system that exhibits high mismatch tolerance for the detection of indel sites in cancer patients.^119^ This platform targets a large number of indel sites in acute myeloid leukemia (AML) and minimal residual disease (MRD), at the same time, the potential for further integration with microfluidic chip systems is highlighted in the study.^119^

A novel chip based on surface-enhanced Raman spectroscopy (SERS)-activated silver nanorod (AgNRs) combines catalytic hairpin assembly (CHA) and CRISPR-Cas13a activity, enabling the sensitive detection of SARS-CoV-2 RNA.^120^ This system, operating as a one-pot reaction, integrates the strong signal amplification effect of CHA with the high specificity of CRISPR-Cas13a, achieving exceptional sensitivity that is 500 times more sensitive than traditional CRISPR/Cas13a-based methods alone and 15,000 times more sensitive than immunoassays such as ELISA.^120^ In another study, the CRISPR/Cas12a system was integrated with SERS to detect viral DNA (HPV-16, HPV-18, HBV) without requiring amplification.^121^ A SERS-activated nanoarray composed of gold nanoparticles and graphene oxide achieved attomolar sensitivity.^121^ This platform offers a new avenue for highly sensitive, rapid, and amplification-free diagnosis.

The key characteristics of these CRISPR-on-chip systems, showcasing their diverse formats and target analytes have been summarized (Table 1).

**Table 1:** Overview of CRISPR-on-Chip Diagnostic Platforms

platform	CRISPR-Cas system	chip type	target	pathogen	signal output	amplification	sensitivity (LOD)	advantages and disadvantages	source
one-pot chip for gut pathogen detection	CRISPR-Cas12a	polymer-based	DNA	*E. coli*, *P. aeruginosa*, *S. aureus*	fluorescent	RPA	>0.43 cfu/mL	simultaneous detection, good programmability	^39^
smartphone-mediated self-powered biosensor	CRISPR-Cas12a	paper-based	miRNA-141		electrochemical and Colorimetric	free	3.1 aM (electrochemical) and 15 aM (colorimetric)		^100^
PLACID	CRISPR-Cas12a	paper-based	DNA/RNA	SARS-CoV-2 and *E. coli*	fluorescent	LAMP/RT-LAMP	50 copies/μL	smartphone app-mediated readout analysis	^101^
automated digital microfluidics system	CRISPR-Cas12a	digital microfluidics	DNA	*H. pylorii*	fluorescent	RPA	10 copies/rxn	automation, high specifity and sensitiveness, multiple sample processing	^107^
mutaSCAN	CRISPR-Cas12a	digital microfluidics/DL	RNA	SARS-CoV-2 and variants	fluorescent	RT-LAMP	250 copies/mL	increased throughput, extraction-free, inadequate for PoC	^115^
Cas12a-MRVDB	CRISPR-Cas12a	centrifugal microfluidics	RNA	14 viruses (SARS-CoV-2 variants, SARS, MERS, and others)	fluorescent	RT-RPA	1 copy/μL	high multiplexing, ultrasensitivity, unfavorable reagent stability	^111^
harmonized AMIC	CRISPR-Cas13a	centrifugal microfluidics	RNA	12 respiratory bacteria	fluorescent	RAA	10 CFU/mL	all-in-one approach	^112^
LIAMT	CRISPR-Cas12c	centrifugal microfluidics	RNA	SARS-CoV-2 and HIV	fluorescent	RT-RPA	73.4 and 63.9 copies/μL	sample-to-result approach, simple, portable	^113^
DEMA	CRISPR-Cas12a	microwell array chip	DNA	*Cryptococcus*	fluorescent	free	0.5 pM	high performance, deep-learning based analysis	^47^
smartphone-enabled digital CRISPR device	CRISPR-Cas12a		RNA	HIV	fluorescent	RT-RPA	75 copies	smartphone-enabled deep-learning-based image analysis, extremely high cost	^46^
plug-and-play CNT-FET biosensor	CRISPR-Cas12a		cardiac troponin I (cTnI)		fluorescent	free	0.33 fg/mL	unable to detect multiple protein targets, promising for microfluidics field	^116^
MOF–DNA biobarcode-amplified CRISPR microfluidic platform	CRISPR-Cas12a	single-cell microfluidic chip	telomerase		fluorescent	MOF–DNA biobarcode amplification	single-cell level	high sensitivity, single-cell detection, early cancer diagnosis	^117^
CoHIT	CRISPR-Cas12a	microfluidic chip	NPM1 gene c.863_864 4-bp insertions		fluorescence	enzymatic recombinase amplification (ERA)	≤0.01%	rapid (30 min), low-cost, multiplex detection, no WT cross-reactivity	^119^
Cas13a-CHA-SERS	CRISPR-Cas13a	silver nanorods (AgNRs) SERS chip	RNA	SARS-CoV-2	Surface-Enhanced Raman Spectroscopy (SERS)	Catalytic Hairpin Assembly (CHA)	5.18 × 10^2^ copies/mL	ultrasensitive, high specificity, multiplex potential	^120^

### 4.3 CRISPR-on-Chip for Personalized Medicine Applications

CRISPR-on-Chip applications have shown great potential for personalized medicine applications. A promising avenue of research is the rapid identification genetic mutations or variations in patients. To this end, a CRISPR-integrated graphene-based field-effect transistor offered digital detection of a target sequence, relevant for Duchenne muscular dystrophy, within an intact genomic sequence.^122^ Rapid detection of patient-specific mutations can enable cost-effective and accessible hereditary disease panel development or aid in carrier screening. By achieving single-nucleotide variation discrimination in diseases such as sickle cell disease, CRISPR-on-chip platforms have demonstrated great potential for rapid genetic disease screening.^123^ Mutliplex testing can further expand the use of these platforms.^37^ Beyond genetic disease diagnosis or carrier identification, this technology can also be translated into minimal residue disease tracking or cancer driver mutation identification.^124^ Person-centered therapy has transformed medical oncology with targeted therapies significantly improving patient outcomes. A majority of targeted therapy regimens are guided by the genetic composition of patient tumors, and the rapid identification of cancer driver genes can aid in personalized disease modeling. For example, CRISPR-on-chip platforms have been used to identify EGFR 19del mutations^125^ and TP53 hotspot mutations.^126^ In addition to identifying driver mutations or variations in patient samples, CRISPR-on-chip can also enable patient-specific drug screening, which can potentially transform therapeutic strategies. By coupling microfluidic organoid cultures with CRISPR biosensors, real-time molecular responses to therapy can be monitored.

## 5 Current Limitations and Future Perspectives

Molecular diagnostics has been transformed by CRISPR-on-chip technology, which combines the special sensitivity of CRISPR-Cas with the possibility of automation and miniaturization in microfluidic systems. Although CRISPR-Cas-integrated microfluidic chip-based platforms developed in recent years have demonstrated impressive sensitivity and portability, scalability, real-time analysis, and multiple target detection,^50,90,122^ the need for improvement in clinical applications is still critical, and urgent development needs continue in these areas.^127^ Many platforms still require off-chip steps such as extraction or purification, which can introduce the risk of contamination and increase expenses.^128^ Fully automated CRISPR-on-Chip platforms that can perform sample processing and purification, signal/isothermal amplification, and final detection in a single step are user-friendly and require minimal equipment, which increases their importance in the future.^36,127^

To overcome the limitations addressed and to satisfy the full potential of CRISPR diagnostics at the PoC level, microfluidic technologies offer a more effective alternative to traditional methods while reducing cost, time, and contamination risks in field tests with automated, miniaturized processes, efficient fluid manipulation, and biosensor integration.^19,92^ Integrating microfluidic systems with CRISPR-Cas platforms provides sensitive and holistic detection of the relevant deficiencies.^79^ In this context, many microfluidic diagnostic platforms have been developed, and electronic and digital microfluidic systems constitute an essential part of the diagnostic field.^13,122^ At the core of electronic microfluidic platforms, transducers convert biochemical signals into electrical outputs by utilizing the enzymatic reaction of biomolecules immobilized on a specific surface.^13,79,122^ These platforms provide fast, selective, economical, user-friendly, and real-time monitoring in diagnostics.^79^ Digital platforms are based on advanced strategies such as droplet microfluidics and microchamber arrays and offer high sensitivity, minimum reagent usage, and strong integration capability.^79^

For many platforms, achieving low LOD without introducing preamplification steps has remained a core bottleneck of CRISPR-on-Chip platforms. Recent platforms have demonstrated LODs at the femtomolar range in identifying target regions with single-nucleotide accuracy in unamplified genomic samples.^122^ CRISPR diagnostics have not only shown high versatility and programmability,^129^ but platforms have also demonstrated clinical-grade sensitivity and specificity comparable to RT-PCR (100% negative agreement, 95% positive agreement).^128^

Multiple detection platforms that enable simultaneous analysis of numerous pathogens or nucleic acid/non-nucleic acid–based disease biomarkers on a single platform will become the main focus of future studies in parallel with the developments in microfluidic technology. In addition, more effective programming of the Cas enzyme with combined crRNA and gRNA configurations is critically important.^38^ The current trend also seeks to combine CRISPR detection with nanomaterial-based signal amplification (plasmonic nanoparticles, quantum dots, and electrochemical biosensors) to enable attomolar detection limits for low-concentration targets.^36,102^ Studies have proposed using pressure/vacuum systems to generate droplets efficiently, minimizing sample consumption in multiplexed assays.^130^ Multiplexed CRISPR platforms, such as CARMEN, have been developed with the ability to simultaneously differentiate between 169 human-associated viruses using nanoliter droplets, demonstrating high multiplexing capabilities while decreasing cost per test by 300-fold.^37^ Such platforms have been further enhanced with microfluidics integration (mCARMEN) and achieved up to 100% sensitivity.^131^

Although CRISPR platforms exhibit undeniable qualities such as high specificity, modularity, and programmability, may prove suboptimal in PoC applications.^79^ The main barriers to the widespread use of existing CRISPR-Dx systems are a lack of integration, long preparation time, low detection rate, and low sensitivity.^115^ On-chip control and standardization require precise control of reaction temperature, flow rate, and reagent mixing. Achieving such standardization in mass production remains challenging without the development of standardized fabrication protocols. Moreover, numerous infectious diseases continue to spread undetected in many low and middle-income countries (LMICs) due to a lack of easily accessible diagnostic tools at the primary healthcare level.^90^ Particularly in low-resource rural settings with limited access to resources, cold-chain requirements create major hurdles in widespread adoption. Laboratory-based diagnostic systems may eventually be replaced by portable, inexpensive, and cutting-edge CRISPR-on-Chip platforms with sensitivity and specificity comparable to PCR.^37,89,127^ These platforms allow for real-time infectious disease monitoring and can be positioned as a proactive tool in managing possible outbreaks.^127^ They also have the potential to provide early diagnosis of cancer and some genetic disorders.

Integrating AL- and DL-based algorithms into CRISPR-on-Chip systems can play an important role in the future of CRISPR-on-Chip diagnostics.^45^ Off-target risks have been considered one of the most important challenges associated with CRISPR-based platforms. Data-driven sgRNA models have been used to profile (and predict) off-target activity of sgRNA to optimize Cas9 activity,^132^ Similarly, DL-enhanced tools have been developed to predict on-target gRNA activity, enhancing gRNA efficiency prediction.^133^ Such applications can be used for on-chip platforms to maximize on-target activity while minimizing off-targets, increasing the sensitivity and specificity of diagnostics platforms.

Besides mitigating off-target effects, AI/ML-based applications can also be used for automated microfluidic design and control. While droplet-based microfluidics holds great potential for screening purposes, there is limited predictive understanding of droplet generation.^134^ As a result, processes require time-intensive and expensive fabrication processes. ML has enabled the design automation of droplet-based microfluidic platforms, which can potentially facilitate the widespread and rapid integration into CRISPR-on-chip platforms.^135^ Such ML frameworks can be leveraged to optimize droplet generators and flow regimes, expediting diagnostic-platform development.

AI/ML integration can also improve analysis processes. Integrated with smartphone image processing and analysis systems, it can increase fluorescence reading accuracy and automate data interpretation by reducing false positive and negative results. Such pipelines can offer significant advantages in infectious disease control during outbreaks. Given the growing interest and success of smartphone-based DL-enhanced readout interpretations, streamlined workflows have become feasible.^136^ In addition, integrating cloud-based systems can enable decentralized PoC healthcare management and real-time patient monitoring. Standardization in sample preparation, device calibration, and result interpretation processes will be a priority of discussion topics in the future. Collaboration between industry, academia, and regulatory agencies will be critical for this technology to reach clinical validity.

## 6 Conclusions

Integrating CRISPR-Cas-based diagnostic platforms to microfluidic chips, CRISPR-on-chip technology will enable portable, quick, and ultrasensitive PoC diagnosis. They offer some crucial benefits, e.g., low cost, low infrastructure requirements, and compatibility with cutting-edge technologies in demand, so they have revolutionized personalized medicine and infectious disease diagnostics. However, more efficient sample manipulation techniques, signal amplification strategies, and data analysis methodologies based on AL/ML must be developed to raise the adoption of this technology in clinics. As the sensitivity and scalability of CRISPR-on-Chip platforms continue to increase, it is expected to become a strong alternative to conventional diagnostic platforms.
